# Capacity of Women for Bearing Pain

**Published:** 1896-09

**Authors:** 


### Capacity of Women for Bearing Pain.

It is obviously a somewhat difficult matter to estimate the degree of resistance to painful impressions in man and woman respectively, or even between individuals of the same sex. Some measure of success has, it is true, attended attempts to express in figures comparative sensitiveness to tactile impression and in respect to the auditory and olfactory functions. There, however, we are dealing with tangible stimuli, which can themselves be titrated, so to speak, before being employed. It is quite otherwise with the subjective phenomenon which we call pain. Pain, indeed, is a reaction which varies more with the idiosyncrasy of the individual than with the nature or intensity of the excitant. The sensations associated with the violent abduction of a tooth are always disagreeable, but it is difficult to resist the conclusion that, other things being the same, the intensity of the pain varies within very wide limits. For purposes of comparison we are obliged to assume what we may call a standard intensity of pain, and, proceeding on that basis, M. J. Finot feels justified in asserting that women are more resistant than men. When under the stimulus of emulation, women, he tells us, are capable of developing a will power far in excess of that attainable by her hardier partner in the "struggle for life." For instance, in experiments with the electric current, the female subjects were able to bear as much as 230 volts, compared with forty or fifty volts, which was all the men would put up with. According to this observer, this capacity of resistance to pain constitutes a valuable attribute in the struggle for life. Woman's intelligence being approximately equal to that of man this extraordinary will-power confers an indisputable superiority over man. The author clinches his argument by pointing out, on biological data, that femininity of sex is the result of a superabundance of vitality, of a richness of nutrition, rather than of any arrest of development, as we have hitherto complacently supposed. The caterpillars of moths and butterflies become of the male sex when subjected to starvation *regime*, and in poor and miserable countries, the preponderance of boys over girls is very marked, just as twins, who have to compete for the maternal nourishment, and are comparatively less favorably situated from a nutritive point of view, are, he says, usually of the male sex. Everywhere and always, concludes M. Finot, nature shows a preference for the female sex, a preference, it may be added, which men have universally endorsed. The ladies ought to vote a medal to M. Finot for his contribution to their emancipation on logical and biological grounds.—*Med. Press and Circular.*

